# Osseointegration of zirconia implants: an SEM observation of the bone-implant interface

**DOI:** 10.1186/1746-160X-4-25

**Published:** 2008-11-06

**Authors:** Rita Depprich, Holger Zipprich, Michelle Ommerborn, Eduardo Mahn, Lydia Lammers, Jörg Handschel, Christian Naujoks, Hans-Peter Wiesmann, Norbert R Kübler, Ulrich Meyer

**Affiliations:** 1 Department for Cranio- and Maxillofacial Surgery, Heinrich-Heine-University Duesseldorf, Germany; 2 Department for Prosthetic Dentistry, Section of Materials Sciences, Johann Wolfgang Goethe University Frankfurt, Germany; 3 Department for Operative and Preventive Dentistry and Endodontics, Heinrich-Heine-University Duesseldorf, Germany; 4 Department for Cranio- and Maxillofacial Surgery, Westfalian Wilhelms-University Muenster, Germany

## Abstract

**Background:**

The successful use of zirconia ceramics in orthopedic surgery led to a demand for dental zirconium-based implant systems. Because of its excellent biomechanical characteristics, biocompatibility, and bright tooth-like color, zirconia (zirconium dioxide, ZrO_2_) has the potential to become a substitute for titanium as dental implant material. The present study aimed at investigating the osseointegration of zirconia implants with modified ablative surface at an ultrastructural level.

**Methods:**

A total of 24 zirconia implants with modified ablative surfaces and 24 titanium implants all of similar shape and surface structure were inserted into the tibia of 12 Göttinger minipigs. Block biopsies were harvested 1 week, 4 weeks or 12 weeks (four animals each) after surgery. Scanning electron microscopy (SEM) analysis was performed at the bone implant interface.

**Results:**

Remarkable bone attachment was already seen after 1 week which increased further to intimate bone contact after 4 weeks, observed on both zirconia and titanium implant surfaces. After 12 weeks, osseointegration without interposition of an interfacial layer was detected. At the ultrastructural level, there was no obvious difference between the osseointegration of zirconia implants with modified ablative surfaces and titanium implants with a similar surface topography.

**Conclusion:**

The results of this study indicate similar osseointegration of zirconia and titanium implants at the ultrastructural level.

## Background

Dental implants are a well-accepted and predictable treatment modality for the rehabilitation of partially and completely edentulous patients. Ten-year survival rates > 95% and 15-year survival rates > 92% have been reported [1]. To achieve long-term success, rigid fixation of the implants within the host bone site is required [2]. This biological concept of osseointegration was first introduced by Branemark et al. in the 1960s [3]. Since its introduction the term osseointegration has been successively redefined, the common denominator being an inanimate metallic structure anchored long-term in living bone under functional loading [4]. Nowadays, commercially pure (cp) titanium and its alloys are the materials most often used in implant manufacturing because of their excellent biocompatibility, favourable mechanical properties and well-documented beneficial results. When exposed to air titanium immediately develops a stable oxide layer which forms the basis of its exceptional biocompatibility. The properties of the oxide layer, i.e. its chemical purity and surface cleanliness, are of great importance for the biological outcome of the osseointegration of titanium implants [5]. According to Albrektsson et al., the quality of the implant surface is one major factor that influences wound healing at the implantation site and subsequently affects osseointegration [6]. In recent years, much effort has been made to improve implant anchorage in bone tissue by modifying the surface characteristics of titanium implants. Various studies have demonstrated that the success of integration of implants into bone tissue correlates positively with a special roughness of the implant surface [7]. Another advantage of a roughened titanium surfaces is a shorter healing period and the option of utilizing shorter implants, still with a good long-term prognosis because of the better bone anchorage [8]. Therefore, many surface modifications of titanium implants have been developed to achieve better osseointegration (machined, plasma-sprayed, grit blasted and/or acid etched).

In recent years, high-strength ceramics have become attractive as new materials for dental implants. They are considered to be inert in the body and exhibit minimal ion release compared to metallic implants. Zirconium oxide partially stabilized with yttrium (yttrium-stabilized tetragonal polycrystals [Y-TZP]) appears to offer advantages over aluminium oxide for dental implants due to its higher fracture resilience and higher flexure strength [9]. Zirconia ceramics have also been successfully used in orthopedic surgery to manufacture ball heads for total hip replacements and this is still the current main application of this biomaterial [10,11].

Up to now, only few studies have investigated the osseointegration of dental implants made of zirconia ceramics. As the surface topography of zirconia implants seems to influence the bone implant interface, the aim of this study was to investigate the osseointegration of zirconia implants with modified ablative surfaces at the ultrastructural level.

## Materials and methods

### Experimental animals

Twelve minipigs (> 5 years, average body weight 66.5 kg) were used in this study. The investigation was approved by the Animal Ethics Committee of the University of Duesseldorf. The animals were kept in small groups in purpose-designed sties and fed on a standard diet. Twelve hours before surgery, the animals received no more feed while water was accessible ad libitum.

### Implant system

Twenty-four screw-type zirconia implants (yttrium-stabilized tetragonal polycrystals) with roughened surfaces produced by acid etching were compared to 24 implants of commmercially pure titanium with acid etched surfaces. Implants were supplied by Konus Dental Implants (Bingen, Germany). All implants had the same geometry with a standardized diameter of 3.5 mm and a length of 9 mm.

### Surgical procedure

All surgery was performed under sterile conditions in a veterinary operating theatre. The animals were sedated by intramuscular injection (10 mg/kg) of ketamine (Ketavet^®^, Pfizer, Karlsruhe, Germany), 1 ml atropine (Atropinsulfat Braun^®^, Braun, Melsungen, Germany) and 5 mg/kg azaperone (Stresnil^®^, Janssen-Cilag, Neuss, Germany). Anaesthesia was induced with an intravenous bolus of 3–5 ml thiopental (Thiopental inresa^®^, Inresa Arzneimittel, Freiburg, Germany) followed by intubation and maintenance of anaesthesia by inhalation of 1.5% isoflurane. For analgesia, animals received 0.5 ml piritramide (Dipidolor^®^, Janssen-Cilag, Neuss, Germany). In the areas exposed to surgery 5 ml of local anaesthesia [articain hydrochloride, (Ultracain^® ^DS, 1:200.000), Aventis, Frankfurt, Deutschland] was injected. The tibias were exposed by skin incisions and via fascial-periosteal flaps. Thereafter, four implants were inserted into the tibia. The implant sites were sequentially enlarged with two drills according to the standard protocol of the manufacturer. Implants were inserted using continuous external sterile saline irrigation to minimize bone damage caused by overheating. At the surgical site, the skin and the fascia-periosteum were closed in separate layers with single resorbable sutures (Vicryl^®^2-0, Ethicon, Norderstedt, Germany). Perioperatively, the animals received amoxicillin (10 mg/kg KG) (Duphamox LA^®^, Fort Dodge, Würselen, Germany) as antibiotic and carproven p.o. (4.4 mg/kgKG) (Rimadyl^®^, Pfizer, Karlsruhe, Germany) as antiphlogistic medication for three days. The animals were inspected after the first few postoperative days for signs of wound dehiscence or infection and, thereafter, weekly to assess general health. After 1, 4 or 12 weeks animals were sacrificed (4 minipigs each) with an overdose of pentobarbital (Eutha 77^® ^ad us. vet, Essex Pharma, München, Germany) given intravenously. Following euthanasia, tibia block specimens containing the implants and surrounding tissues were dissected from all of the animals. The block samples were sectioned by a saw to remove unnecessary remnants of bone and soft tissue and were prepared for the subsequent investigations.

### Scanning electron microscopy (SEM)

**Figure 1:**
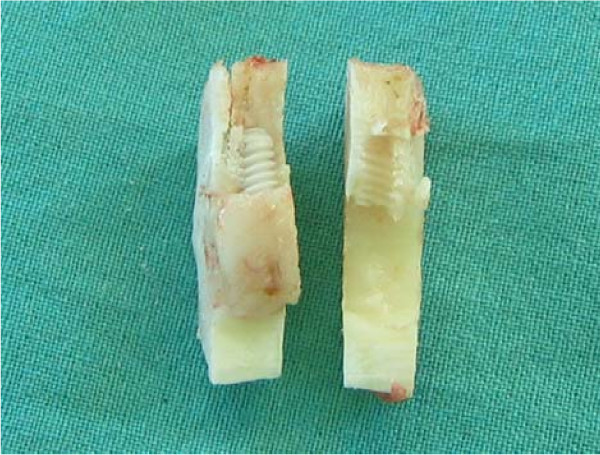
Dissection of the block samples into two halves.

Utilizing the fracture technique [12] the block samples were dissected into two halves (Figure 1). Samples containing the implants were used for scanning electron microscopy (SEM), the corresponding bone samples for EDX analysis. After fixation in 4% glutaraldehyde, the specimens were dehydrated in a graded series of ethanol and critical-point dried. Samples were then sputter-coated with gold and examined under a JEOL 6300F (JEOL, Eching, Germany) high-resolution field emission scanning electron microscope.

## Results

**Figure 2:**
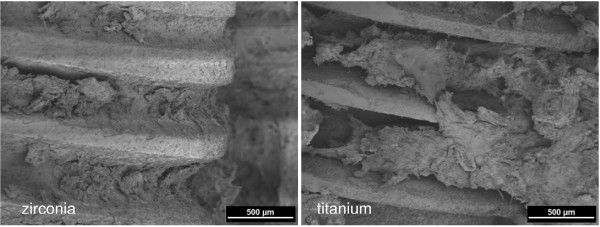
**Bone matrix adherent to the implant surface after one week healing time.** Zirconia implant (left), titanium implant (right) (2 kV, magnification 50-fold).

**Figure 3:**
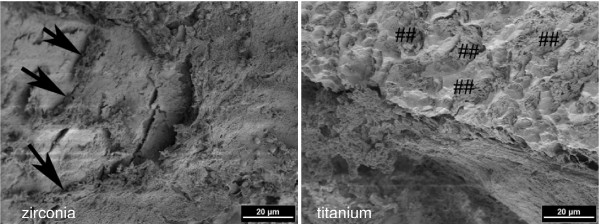
**One week healing time: dense fibrinous and collagenous bone matrix in direct contact with the zirconia implant surface (arrows) (left).** Anchorage of bone matrix in the cavities of the titanium implant surface (##) (right) (2 kV, magnification 1000-fold).

**Figure 4:**
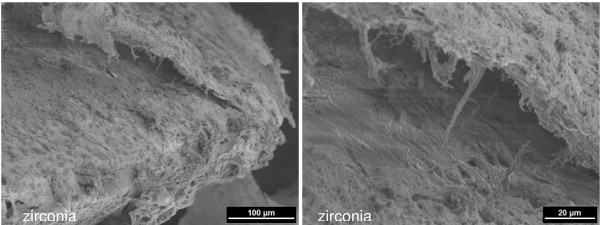
**4 weeks healing time: zirconia implant well covered with mineralized tissue, osteoid, and dense matrix (left) (2 kV, magnification 250-fold).** Enlarged detail: fibrous matrix in the artifical gap resulting from the dissection of the specimen (right) (2 kV, magnification 1000-fold).

**Figure 5:**
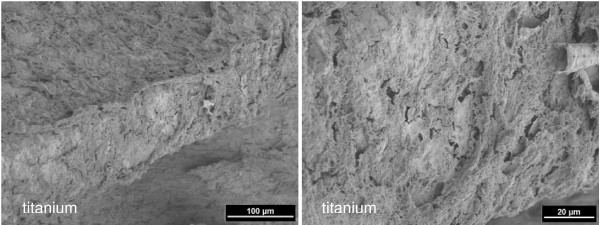
**4 weeks healing time: coverage of the titanium implant with mineralized tissue and dense bone matrix (left) (2 kV, magnification 250-fold).** Enlarged detail (right) (2 kV, magnification 1000-fold).

**Figure 6:**
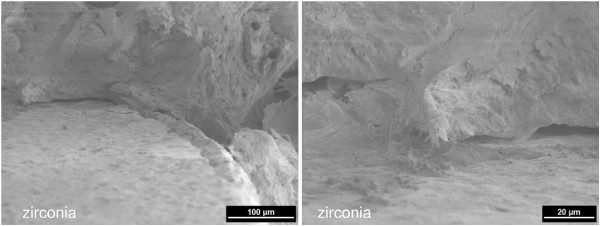
**12 weeks healing time: mature bone in direct contact with the surface of the zirconia implant (left) (2 kV, magnification 250-fold).** Enlarged detail: artificial gap resulting from the dissection of the specimen (right) (2 kV, magnification 1000-fold).

**Figure 7:**
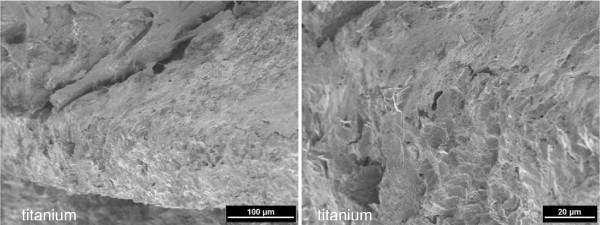
**Ingrowth and anchorage of mature bone on the surface of the titanium implant (left) (2 kV, magnification 250-fold).** Enlarged detail (right) (2 kV, magnification 1000-fold).

All surgical procedures were performed without complications and the animals recovered well after surgery. Daily investigation showed no clinical signs of infection during the observation period. Scanning electron microscopy demonstrated that, already after one week, osseous matrix adhered to the implant surfaces of the zirconia and titanium implants (Figure 2). At higher magnification, differences in matrix composition were observed. On the zirconia implants, a dense matrix mainly consisting of fibrinous and collagenous filaments in combination with corpuscular and cellular components was found in direct contact with the ZrO_2 _surface (arrows in Figure 3 left). On the titanium implants, less osseous matrix was seen on the surface. Extracellular matrix was anchored particularly in the cavities of the titanium surface (Figure 3 right). After 4 weeks, intimate contact with bone cells embedded in a mineralized collagen-rich extracellular matrix was present on both titanium and zirconia implant surfaces. Although a slightly higher degree of mineralization was detected on the titanium surface, there were no significant differences between the bone-implant interfaces of the two implant materials (Figures 4 and 5). After 12 weeks, successful osseointegration of the zirconia as well as the titanium implants was visualized at the ultrastructural level. Scanning electron microscopy showed newly developed bone integrated at the implant surfaces and confirmed the intimate contact of the mature lamellar bone with the zirconia and titanium surfaces. No interposition of an interfacial layer or foreign-body reaction was detected in any of the samples examined (Figures 6 and 7).

## Discussion

In the study presented, osseointegration of zirconia implants with a modified ablative surface was investigated at the ultrastructural level and compared to titanium implants with a similar surface structure. One week after implantation, SEM analysis demonstrated a firm attachment of collagen-rich extracellular bone matrix to both implant surfaces. Three weeks later, both titanium and zirconia implant surfaces had made intimate contact with mineralized tissue, osteoid, and dense collagen-rich extracellular matrix. After 12 weeks, ultrastructural evidence of a successful osseointegration of both implant systems was found.

Insights into cellular processes occurring at the bone-implant interface are important for the understanding of biocompatibility and osseointegration. The resulting knowledge will contribute to the production and testing of biomaterials with specific and desired biological responses [13,14]. According to Büchter and co-workers [15], probe processing by sample fracturing for the SEM investigation is indicative of the fact that the bonding strength between the implant and the adjacent bone layer seems to reach bonding values in the bone itself. After one week, firm anchorage of the extracellular matrix at the complete titanium surface was evident particularly at the interdigitation of titanium peaks on the implant demonstrating that implant anchorage is dependent on surface topography [16]. Rougher surfaces are predicted to promote mainly osteoconduction by increasing available surface area for fibrin attachment and by providing surface features with which fibrin could become entangled [17]. The results of the present study are in agreement with Simmons et al. [18] who investigated the early healing response of porous-surfaced implants compared to Ti plasma-sprayed implants inserted in rabbit femoral condyles. After four days, the necrotic bone that had been created during surgery had been resorbed and a well-defined interface zone had formed adjacent to both investigated implant designs. Scanning electron microscopical analysis revealed a fibrinous and collagenous matrix extensively interdigitated with the three-dimensional interconnected porous structure in contrast to the plasma-sprayed implants. After 8 days of healing, coverage and interdigitation of the healing tissue had increased on both surfaces. However, the matrix around porous-surfaced implants appeared more dense and extensive compared with plasma-sprayed implants. After 16 days, both implant surfaces were well covered and extensively integrated into a mixture of mineralized tissue, osteoid, and dense matrix. No significant differences in strength and stiffness of attachment between the two implant designs were detected at this time point. Similar results are shown by Büchter et al. [15]. They demonstrated complete osseointegration of differently loaded titanium implants after 28 days by SEM observation.

A recent SEM evaluation of the human bone-oxidized titanium interface showed ingrowth and anchorage of bone independant of the pore size. In contrast to previously published studies, intimate bone contact was present in pores with a diameter of less than 2 μm [19].

Only one recent study investigated the bone implant interface of titanium and zirconia implants with different surface modifications [9]. Sennerby et al. detected no significant differences after six weeks of healing time. Ultrastructural analysis revealed intimate contact of bone tissue with the material surfaces in their study, indicated by the observation of fracture of the bone rather than separation of the interface. These results are in agreement with our findings presented in this study, confirming that the surface texture positively influences bone integration.

## Conclusion

The results of the present study showed no significant differences between bone tissue response to a surface-roughened zirconia implant and a titanium implant with a similar surface structure.

## Competing interests

The authors declare that they have no competing interests.

## Authors' contributions

UM, CN conceived the study design and performed surgery. EM, LL, HPW carried out the SEM analysis and drafted the manuscript. RD participated in the design of the study, performed surgery and wrote the manuscript. HZ, MO, JH, NRK participated in the early preparation of the manuscript and contributed to write the revised version of the article. All authors read and approved the final manuscript.
